# Risk Factors for Mortality in Patients with Septic Acute Kidney Injury in Intensive Care Units in Beijing, China: A Multicenter Prospective Observational Study

**DOI:** 10.1155/2014/172620

**Published:** 2014-07-07

**Authors:** Xin Wang, Li Jiang, Ying Wen, Mei-Ping Wang, Wei Li, Zhi-Qiang Li, Xiu-Ming Xi

**Affiliations:** ^1^Department of Critical Care Medicine, Fu Xing Hospital, Capital Medical University, Beijing 100038, China; ^2^Department of Surgical Intensive Care Units, Hepatobiliary Surgery and Liver Transplant Center, Beijing YouAn Hospital, Capital Medical University, Beijing 100069, China; ^3^Center for Infectious Diseases, Beijing YouAn Hospital, Capital Medical University, Beijing 100069, China; ^4^Department of Critical Care Medicine, Hospital affiliated to Hebei United University, Tangshan 06300, China

## Abstract

*Objective*. To discover risk factors for mortality of patients with septic AKI in ICU via a multicenter study.* Background*. Septic AKI is a serious threat to patients in ICU, but there are a few clinical studies focusing on this.* Methods*. This was a prospective, observational, and multicenter study conducted in 30 ICUs of 28 major hospitals in Beijing. 3,107 patients were admitted consecutively, among which 361 patients were with septic AKI. Patient clinical data were recorded daily for 10 days after admission. Kidney Disease: Improving Global Outcomes (KDIGO) criteria were used to define and stage AKI. Of the involved patients, 201 survived and 160 died.* Results*. The rate of septic AKI was 11.6%. Twenty-one risk factors were found, and six independent risk factors were identified: age, APACHE II score, duration of mechanical ventilation, duration of MAP <65 mmHg, time until RRT started, and progressive KIDGO stage. Admission KDIGO stages were not associated with mortality, while worst KDIGO stages were. Only progressive KIDGO stage was an independent risk factor.* Conclusions*. Six independent risk factors for mortality for septic AKI were identified. Progressive KIDGO stage is better than admission or the worst KIDGO for prediction of mortality. This trial is registered with ChiCTR-ONC-11001875.

## 1. Introduction

Globally, the incidence of acute kidney injury (AKI) has increased steadily in recent years [1–4]. AKI is commonly seen in critically ill patients in ICU [5, 6] and contributes to the failure of other organs and systems in such patients [7]. The duration of AKI can be used to predict disease severity and outcome [8] although even transient AKI is linked to increased mortality [9]. The risk of death in AKI patients shows an incremental increase corresponding to disease stage [10]. Known risk factors of AKI include sepsis, critical illness, circulatory shock, burns, trauma, cardiac surgery, chronic diseases (heart, lung, and liver), major noncardiac surgery, and nephrotoxic drugs [11].

The cause of AKI in critically ill patients is usually multifactorial; however, sepsis is one of the leading causes of AKI, contributing to more than half of all reported cases [12–14]. The mechanism of sepsis-induced AKI is a complex combination of factors such as vascular and glomerular thrombotic processes, inflammation, and shock and is distinct from nonseptic AKI [15–18]. Thus, the clinical presentation, outcome, and responses to therapy may differ between septic and nonseptic AKI. Septic AKI is coupled with a significantly increased risk for hospital death, even after adjustment for relevant covariates [19]. However, only a limited number of clinical studies focusing on septic AKI in ICUs have been reported [19–23]. Thorough investigation is urgently required to reveal the epidemiology, pathophysiology, clinical features, and, more importantly, effective therapeutic measures for this disorder to reduce its high mortality.

This study aimed to identify risk factors for mortality in ICU patients with septic AKI and to evaluate the use of the KDIGO staging system for the prediction of prognosis in this group of patients, via a multicenter clinical study.

## 2. Material and Methods

This observational multicenter study was a retrospective analysis of prospectively collected data from patients in 30 ICUs of 28 major hospitals in Beijing between March 1 and August 31, 2012, as a part of the BAKIT (Beijing AKI Trail) study. Study subjects included all adult patients (age ≥ 18 years) admitted consecutively to the ICU and who received care in the ICU for at least 24 hours. Only the initial ICU admission was considered in this study. The following patients were excluded from the study: patients with preexisting end-stage chronic kidney disease, those already on RRT before admission to ICU, and those who had received kidney transplantation in the previous 3 months.

This study was approved by the Institutional Review Board of the Ethics Committee of the lead study center (Fu Xing Hospital, Capital Medical University, China), which waived the requirement for informed consent for this observational survey. Patient records/information were anonymized and deidentified prior to analysis.

### 2.1. Case Identification

Nine hundred and seventeen patients diagnosed with sepsis were identified [24]. AKI severity was classified according to the KDIGO guidelines (Kidney Disease: Improving Global Outcomes) [11], as follows: AKI is defined by an increase in serum creatinine (SCr) by ≥26.5 *μ*mol/L within 48 hours or an increase of SCr of ≥1.5 times over baseline (which is known or presumed within the prior 7 days) or urine volume <0.5 mL/kg/h for 6 hours. AKI is staged for severity (3 stages) based on the changes in SCr and urine volume. Patients were staged according to SCr or urine output or both, with the criteria leading to the highest stage being used. Baseline SCr was the last value within the preceding year. For patients without these values or without renal failure, baseline SCr was estimated by the Modification of Diet in Renal Disease (MDRD) equation [25], assuming a glomerular filtration rate of 75 mL/min/1.73 m^2^ [6]. For patients with chronic renal failure but not on dialysis, the initial SCr value on admission was used as the baseline value [6].

### 2.2. Data Collection

A uniform case report form (CRF) was used to collect data. Standard demographic, clinical, and laboratory data collected in the ICU included age, sex, dates and source of admission, BMI, blood pressure, duration of ICU stay, comorbidities, nonrenal organ failures, daily fluid input and output, and serum creatinine. The use of interventions, such as RRT, mechanical ventilation, loop diuretic therapy, and vasoactive agents, was also recorded.

Severity of illness was assessed by the Acute Physiology and Chronic Health Evaluation (APACHE) II score and Sequential Organ Failure Assessment (SOFA) scores, which were calculated based on the worst variables recorded during the first 24 hours after ICU admission to evaluate patient status [26, 27]. AKI severity was evaluated by KDIGO staging. Preexisting comorbidities were diagnosed based on International Classification of Diseases (ICD-10). For all patients included in the study, a thorough follow-up was conducted for the first 10 days after ICU admission. Patient status, laboratory data, interventions, and KDIGO stages were recorded daily. End points of this study included death or being transferred out of the ICU.

### 2.3. Definitions

**Figure 1:**
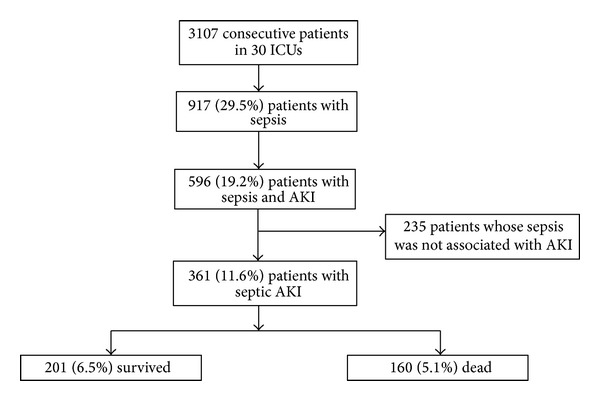
Study protocol flowchart.

Septic AKI was defined as sepsis-associated AKI [20, 28, 29], which meant that sepsis was associated with development and progression of AKI, so the patients (*n* = 235) with sepsis whose sepsis was not associated with AKI were excluded (Figure 1). We defined sepsis according to the American College of Chest Physicians and the Society of Critical Care Medicine(ACCP/SCCM) consensus [24, 30]. Based on this consensus, SIRS is defined as temperature >38°C or <36°C, heart rate >90/min, respiratory rate >20/min or PaCO2 <32 mmHg, and white blood cell count >12,000/mm^3^ or <4,000/mm^3^ or with >10% bands. Sepsis was defined as a condition in which the patient met the criteria for SIRS and presented with either a documented or suspected infection. Admission KDIGO refers to the KDIGO stage on the first day of admission, while worst KDIGO refers to the worst KDIGO stage reached by a patient during their ICU stay. ICU-acquired AKI was defined as the development of AKI at 24 hours or more after admission, with the absence of AKI prior to admission. Progressive AKI was defined as patients reaching a higher KDIGO stage compared with the admission KDIGO stage at any time during their ICU stay. Vasoactive agents used in this study included epinephrine, norepinephrine, dopamine, and dobutamine. Large-dose vasopressor was defined as norepinephrine or epinephrine administered at a dose of >0.1 *μ*g/kg/min, or dopamine or dobutamine administered at a dose of >15 *μ*g/kg/min, or any two or more drugs in combination. Hospital acquired infection was defined as the development of an infection within 48 hours after hospital admission, which was not presented or incubating at the time of admission to the hospital.

### 2.4. Statistical Analysis

SPSS software (version 15.0) was used for data analysis. All variables were tested for normal distribution using the Kolmogorov-Smirnov test. Normally or near normally distributed variables are presented as means and SD, nonnormally distributed continuous data are presented as medians and interquartile ranges (IQR). Student's *t*-test was used for analysis of continuous variables. Mann-Whitney *U* test was used for nonnormally distributed variables. Categorical variables were compared with the chi-square test or Fisher's exact test. A 2-tailed *P* < 0.05 was considered statistically significant. Logistic regression was used to analyze risk factors for mortality. All variables with a *P* value <0.001 were included in the multivariate model. Backward selection based on the likelihood ratio test was used to select the final multivariate model for risk factors of mortality.

## 3. Results

### 3.1. Patient Characteristics

During the 6-month study period, a total of 3,107 patients were admitted to the 30 ICUs involved in this study, of which 29.5% (917/3,107) were diagnosed with sepsis. Of these patients, 39.4% (361/917) of patients were diagnosed with septic AKI; among which 55% (201/361) of patients survived and 44.4% (160/361) died. The rate of septic AKI among all subjects was 11.6% (361/3,107) (Figure 1). The average age was 70.54 ± 16.04 years, and 64.0% were male. Average BMI was 23.16 ± 3.82 and 37.7% were identified as hospital acquired infections. The average first 24 h APPACHE II score in the ICU was 23.59 ± 7.87, and the first 24 h SOFA score was 10.49 ± 5.40. The age, sex, BMI, hospital acquired infection, ways of admission, duration in ICU, nonrenal organ failure, comorbid diseases, and first 24 h APACHE II and SOFA scores in the ICU were compared between survivors and nonsurvivors. The age (*P* < 0.001), hospital acquired infection (*P* = 0.001), surgical admission (*P* = 0.004) and emergency admission (*P* = 0.003), systolic heart failure (*P* = 0.007), malignancy (*P* = 0.031), heart function level IV (*P* = 0.021), first 24 h APACHE II score (*P* < 0.001), and SOFA score (*P* < 0.001) in the ICU were associated with mortality (Table 1).

### 3.2. Disease Progression in the ICU

Disease progression was observed consecutively in the first 10 days after admission to the ICU in this study, and key interventions and parameters were recorded and analyzed. In total, 78.9% of septic AKI patients were on mechanical ventilation, 35.5% of patients needed RRT. Data on mechanical ventilation, fluid balance, hemodynamic data, and duration of vasoactive agent administration, loop diuretic therapy, and RRT were compared between survivors and nonsurvivors. Mechanical ventilation (*P* < 0.001) and its duration (*P* < 0.001), daily fluid balance (*P* = 0.001), duration of MAP <65 mmHg (*P* < 0.001), days on vasopressors (*P* < 0.001) and high-dose vasopressors (*P* < 0.001), RRT (*P* = 0.007), and time interval between ICU admission and RRT initiation (*P* < 0.001) were associated with patient outcomes (Table 2).

### 3.3. KDIGO Stages and Patient Outcome

**Figure 2:**
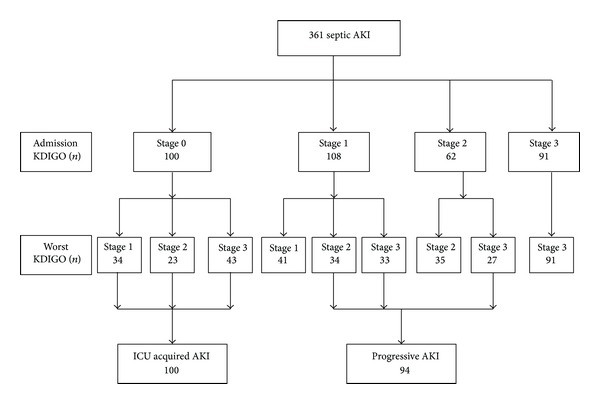
Progression of AKI in ICUs measured by KDIGO stages.

**Figure 3:**
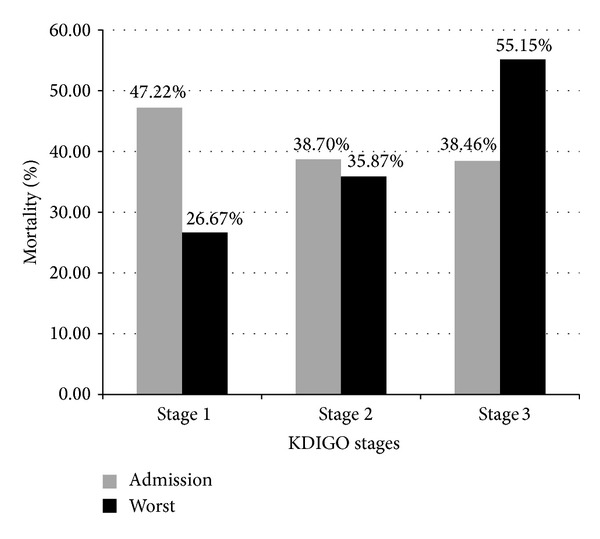
Mortality of septic AKI patients with different admission or the worst KDIGO stages. Gray bars represent mortality rates of patients grouped by admission KDIGO stages; black bars represent mortality rates of the worst KDIGO stages.

During the first 10 days of ICU care, renal function of the patients was evaluated once a day according to KDIGO stage in this study. A flowchart of the progression of AKI in the ICU measured by KDIGO stages is shown in Figure 2. On admission, 27.7% (100/361) of all patients were at KDIGO stage 0, 29.9% (108/361) were at stage 1, 17.2% (62/361) were at stage 2, and 25.2% (91/361) were at stage 3. For the worst KDIGO stages, none of the patients were at stage 0, 20.8% (75/361) of patients were at stage 1, 25.5% (92/361) were at stage 2, and 53.7% (194/361) were at stage 3. Admission KDIGO stages were not linked to patient outcome, while the worst KDIGO stages were. According to our data, patients categorized into KDIGO stages 1, 2, and 3 by the worst KDIGO stages were strongly associated with patient outcome (*P* < 0.001, *P* = 0.038, and *P* < 0.001, resp.). ICU-acquired AKI was not linked to disease outcome (*P* = 0.110). Progressive AKI was associated with mortality (*P* < 0.001) (Table 3). Mortality rates for patients at different admission and the worst KDIGO stages are shown in Figure 3.

### 3.4. Risk Factors for Mortality

To identify possible risk factors for mortality in ICU patients with septic AKI, univariate analysis was performed for all the tested factors with a *P* value <0.05. Multivariate regression analysis was performed for all parameters with a *P* value <0.001 in the univariate analysis. Six independent risk factors were identified: age (OR = 1.025, 95% CI (1.007–1.042), *P* = 0.005), APACHE II score (first 24 h in ICU) (OR = 1.072, 95% CI (1.037–1.109), *P* < 0.001), duration of mechanical ventilation (OR = 1.080, 95% CI (1.008–1.158), *P* = 0.03), duration of MAP <65 mmHg (OR = 1.149, 95% CI (1.032–1.279), *P* = 0.011), time interval between ICU admission and RRT initiation (OR = 1.238, 95% CI (1.115–1.374), *P* < 0.001), and progressive KIDGO stage (OR = 3.374, 95% CI (1.918–5.933), *P* < 0.001) (Table 4).

## 4. Discussion

In this study, we investigated possible risk factors for mortality in critically ill patients with septic AKI via a large, multicenter, and observational study involving 30 ICUs. A total of 21 risk factors and six independent risk factors were identified in a thorough statistical analysis of comparisons between survivors and nonsurvivors among critically ill patients with septic AKI.

Our data showed low mortality among septic AKI patients admitted from the surgical or emergency departments (Table 1). Many surgical patients in the ICU were admitted for routine postoperative care after major operations and were associated with a very low mortality rate. Many patients admitted from emergency departments were in acute conditions, and, after timely interventions in ICU, most of them recovered well.

Mechanical ventilation is a common and important intervention in the ICU. In our study, the use of mechanical ventilation was correlated with increased mortality (*P* < 0.001). This is possibly due to the common complications of mechanical ventilation, such as worsening inflammatory responses, altered systemic hemodynamics, and elevated intrathoracic and intra-abdominal pressure, all of which are involved in the development of AKI [31, 32]. In further analysis, we found that the duration of mechanical ventilation was an independent risk factor for mortality in patients with septic AKI, which may be due to the occurrence of ventilator-associated pneumonia (VAP), one of the leading causes of death in mechanically ventilated patients [33].

Compared with septic AKI survivors, nonsurvivors had greater hemodynamic instability: suffering from longer duration of hypotension (MAP < 65 mmHg), receiving more fluid and vasopressor even large-dose vasopressor (Table 2); in addition, multivariate analysis indicated the duration of MAP <65 mmHg as an independent risk factors for mortality in septic AKI patients (Table 4).

Previous studies have shown that, compared with nonseptic AKI patients, septic ones came with worse hemodynamic instability and required more vasoactive agent use [20, 23, 34]. Lopes and colleagues discovered that extensive use of vasopressors was found in patients with severe AKI and associated with poor prognosis [35]. This is consistent with our data in Table 2.

In critically ill patients, it has been reported that positive fluid balance impaired cardiac function, led to lung injury, and may contribute to the development of AKI, which, in turn, increase mortality [36]. In patients with sepsis, prior report has shown that cumulative positive fluid balance was associated with increased mortality (odds ratio = 1.2) after adjustment for disease severity [37]. In our study, we came to the same conclusion in septic AKI patients (Table 2).

So, it seems that septic AKI patients with a long duration of low MAP required more vasoactive drug use and positive fluid balance, which implied high risk of shock and poor outcome.

RRT is one of the main approaches to the management of AKI. Recently, a multicenter study shown that in the nonsurvival with septic AKI, proportion of receiving RRT was significantly higher than that in the survival [29]. Our findings are consistent with this study: compared with the septic AKI patients who survived, proportion of receiving RRT was significantly higher in who died (43.13% versus 29.35%, *P* = 0.007). Furthermore, we found that there was no significant difference in duration of RRT between survivors and nonsurvivors with septic AKI (*P* = 0.065).

In addition, it is interesting that the time interval between ICU admission and RRT initiation was significantly longer in the patient who died. Moreover, by multivariate analysis, this delay in initiation of RRT was an independent risk factor for mortality (Tables 2 and 4). A large multicenter study about septic AKI came to the same conclusion that the time between ICU admission and start of RRT was significantly longer in the patients with septic AKI and this delay in initiation of RRT was independently associated with hospital mortality [20]. The right time to start RRT is still a topic of debate [38]. Experts recommend beginning RRT earlier, particularly in sepsis where AKI is known to be rapidly progressive [38]. A meta-analysis about timing of RRT clearly favored to begin RRT at early time [39]. In our study, the delay in initiation of RRT associated with mortality might be partly explained that progression of AKI in ICU was also an independent risk factor for mortality (Table 4). Patients with septic AKI who are with progression of AKI in ICU might receive RRT later after ICU admission than patients without progression of AKI. In brief, this observation showed that starting RRT timely is a key factor to reduce the high mortality of patients with septic AKI.

Many previous studies have evaluated AKI in critically ill patients by using the RIFLE classification [40] or AKIN criteria [41] and reported it to be associated with risk for mortality [6, 41–44]. KDIGO criteria is a new scaling system for AKI severity [11] and has been proven to be of prognostic significance [45, 46]. Some studies have indicated that KDIGO classification is better than RIFLE in terms of outcome prediction in certain circumstances [46]. Here we aimed to use KDIGO classification to evaluate critically ill patients with septic AKI. We found that the worst KIDGO stage in the ICU was linked to patient outcome, while no link was identified for the admission classification (Table 3). Furthermore, crude hospital mortality rates showed an incremental increase corresponding to the worst KDIGO stages, but not to the admission classification (Figure 3). This is consistent with previous publications indicating that mortality is not associated with admission RIFLE (risk, 44.7%; injury, 53.2%; failure, 51.0%; *P* = 0.58). However, worst RIFLE is associated within increased 28-day mortality (*P* < 0.01) [21].

Patients with poor admission KDIGO stages can be treated effectively by stage-based management such as hemodynamic monitoring, ensuring volume status and perfusion pressure, monitoring serum creatinine and urine output [11] and early goal-directed therapy (EGDT) [47]. However, later development of AKI or progression to a higher stage of AKI after ICU admission implies poor prognosis [21, 48].

It is interesting that in the worst KDIGO stages (Table 3), we found that only KDIGO stage 3 was associated with a high mortality, while survivors had a greater incidence of KDIGO stages 1 and 2. A multicenter study about septic AKI in Finnish came to the same conclusion; they found that after adjusting for covariates, the worst KDIGO stage 3 was associated with increased risk for 90-day mortality, but stages 1 and 2 were not [29]. It can be explained that although receiving active treatment in ICU, if the severity of septic AKI still progressed to KDIGO stage 3, the mortality would increase significantly. If the worst KDIGO stage of septic AKI only reached stages 1 or 2 in ICU, it would imply a good outcome.

Although the worst KDIGO stages were associated with mortality, they were not independent risk factors, while progressive KDIGO stage was found to be an independent risk factor associated with poor prognosis (Table 4). This is consistent with the results of other septic AKI studies, where progression of AKI has important prognostic implications [21, 49]. This result indicated the necessity of monitoring changes in KDIGO stages when AKI occurred in patients with sepsis in the ICU. On the other hand, ICU-acquired AKI was not a risk factor for mortality in our study (*P* = 0.110) (Table 3) but was an independent risk factor for 28-day mortality in a RIFLE-based study [21]. There were two differences between this study and ours. Firstly, its subject was patients with severe sepsis and septic shock, while the subject of our study was patients with septic AKI. Secondly, this study was a single center study targeting patients from medical ICU, thus limiting the applicability to more heterogeneous populations. In contrast, our study was a multicenter study involving various types of ICUs.

Our study has several limitations. First, baseline creatinine concentration was not measured for all patients; therefore, in such cases this value was estimated using the MDRD equation. Second, use of antibiotics is critical for management of sepsis but was not observed and involved in this study, because this was a substudy of the BAKIT (Beijing AKI Trail) study.

Through a consecutively thorough follow-up for 10 days after ICU admission, we found that the independent risk factors for mortality, except age and APACHE II score, the other four factors were all dynamic observational ones, such as duration (MAP, mechanical ventilation), time interval between admission, and RRT initiation and progression (KDIGO stage), while static factors such as need for RRT and the worst KDIGO stages were not independent risk factors. It suggests that we need to consecutively monitor the conditions of septic AKI patients. Currently, most of observational studies for septic AKI collected clinical data for only one day [19, 28]; therefore the value of these data for predicting the prognostic for septic AKI is limited.

## 5. Conclusion

In summary, via a multicenter observational study, we evaluated the use of KDIGO stages on predicting patient outcome, found twenty-one risk factors such as age, hospital acquired infection, systolic heart failure, and mechanical ventilation, and identified six independent risk factors for mortality in ICU patients with septic AKI, which may help make early and accurate diagnosis and adopting preventive and therapeutic interventions that could reduce mortality rates in patients with septic AKI.

## Figures and Tables

**Table 1:** Patient characteristics.

	Survivors (*n* = 201)	Nonsurvivors (*n* = 160)	*P* value
Age (years; median [IQR])	72 (56–81)	78 (67–83)	<0.001
Gender (male) *n* (%)	131/201 (65.17%)	100/160 (62.50%)	0.264
BMI (mean ± SD)	23.50 ± 3.56	22.73 ± 4.09	0.058
Hospital acquired infection *n* (%)	61/201 (30.35%)	75/160 (46.88%)	0.001
Admission, *n* (%)			
Surgical admission	38/201 (18.91%)	14/160 (8.75%)	0.004
Emergency	83/201 (41.29%)	56/160 (35%)	0.003
Duration in ICU			
Days in ICU (days; median [IQR])	9 (5–16)	9 (5–19)	0.737
Nonrenal organ failure, *n* (%)			
Respiratory failure	124/201 (61.69%)	112/160 (70%)	0.099
Systolic heart failure	11/201 (5.47%)	22/160 (13.75%)	0.007
Hypovolemia shock	17/201 (8.46%)	19/160 (11.88%)	0.282
Septic shock	80/201 (39.8%)	68/160 (42.50%)	0.604
DIC	13/201 (6.47%)	8/160 (5.00%)	0.554
Hepatic failure	12/201 (5.97%)	8/160 (5.00%)	0.500
MODS (nonrenal)	62/201 (30.85%)	65/160 (40.63%)	0.051
Comorbid disease, *n* (%)			
Malignancy	34/201 (16.92%)	43/160 (26.88%)	0.031
Hypertension/CHD	108/201 (53.73%)	94/160 (58.75%)	0.340
Diabetes mellitus	48/201 (23.88%)	33/160 (20.63%)	0.461
CKD without renal failure	11/201 (5.47%)	4/160 (2.50%)	0.160
CKD with renal failure	19/201 (9.45%)	21/160 (13.13%)	0.269
Immunosuppression	9/201 (4.48%)	7/160 (4.38%)	0.586
Organ transplant	5/201 (2.49%)	2/160 (1.25%)	0.47
Heart function level IV	14/201 (6.97%)	23/160 (14.36%)	0.021
APACHEII score, first 24 h in ICU (mean ± SD)	21.41 ± 7.74	26.32 ± 7.15	<0.001
SOFA score, first 24 h in ICU (mean ± SD)	9.42 ± 5.33	11.83 ± 5.21	<0.001

BMI: body mass index, RRT: renal replacement therapy, CKD: chronic kidney disease, ICU: intensive care unit, DIC: disseminated intravascular coagulation, MODS: multiple organ dysfunction syndrome, CHD: chronic heart disease, SOFA: sequential organ failure assessment, APACHE: acute physiology and chronic health evaluation.

**Table 2:** Data on disease progression in the first 10 days after admission to ICU.

	Survivors (*n* = 201)	Nonsurvivors (*n* = 160)	*P* value
Mechanical ventilation			
Patients on mechanical ventilation *n* (%)	145/201 (72.14%)	140/160 (87.5%)	<0.001
Duration on mechanical ventilation (days; median [IQR])	3 (0–7)	6 (3–10)	<0.001
Fluid management			
Duration for positive fluid balance (days; median [IQR])	5 (3–7)	5 (3–7)	0.583
Daily fluid balance (mL/24 h)	654 ± 794	982 ± 1024	0.001
Hemodynamic data			
Duration for MAP < 65 mmHg (days; mean ± SD)	1.50 ± 1.98	2.42 ± 2.60	<0.001
Vasoactive agents			
Days on vasopressors (median [IQR])	1 (0–4)	3 (2–6)	<0.001
Days on large-dose vasopressor (median [IQR])	0 (0–3)	3 (0–5)	<0.001
Loop diuretic therapy (days) (median [IQR])	2 (0–6)	2 (1–5)	0.693
RRT			
Need for RRT *n* (%)	59/201 (29.35%)	69/160 (43.13%)	0.007
Duration of RRT (days; median [IQR])	0 (0–3)	0 (0–2)	0.065
Time interval between admission and RRT initiation (days; median [IQR])	0 (0-1)	0 (0–4)	<0.001

RRT: renal replacement therapy; MAP: mean arterial pressure.

**Table 3:** AKI classified by KDIGO criteria.

	Survivors (*n* = 201)	Nonsurvivors (*n* = 160)	*P* value
KDIGO stage on admission *n* (%)			
Stage 1	57/201 (28.36%)	51/160 (31.88%)	0.271
Stage 2	38/201 (18.91%)	24/160 (15%)	0.202
Stage 3	56/201 (27.86%)	35/160 (21.88%)	0.119
Worst KDIGO stage in ICU *n* (%)			
Stage 1	55/201 (27.36%)	20/160 (12.5%)	<0.001
Stage 2	59/201 (29.35%)	33/160 (20.63%)	0.038
Stage 3	87/201 (43.28%)	107/160 (66.88%)	<0.001
Progress KDIGO stage class *n* (%)			
ICU acquired AKI	50/201 (24.88%)	50/160 (31.25%)	0.110
Progressive AKI	32/201 (15.92%)	62/160 (38.75%)	<0.001

KDIGO: Kidney Disease: Improving Global Outcomes.

**Table 4:** Regression analysis of risk factors for mortality in ICU.

	Univariate analysis		Multivariate analysis	
	OR (95% CI)	*P* value	OR (95% CI)	*P* value
Age (years)	1.026 (1.012–1.041)	<0.001	1.025 (1.007–1.042)	0.005
Hospital acquired infection	2.025 (1.314–3.120)	0.001		
Nonrenal organ failure				
Systolic heart failure	2.754 (1.293–5.866)	0.009		
Comorbid disease				
Malignancy	1.748 (1.050–2.911)	0.032		
Heart function IV	2.242 (1.114–4.516)	0.024		
APACHE II score	1.092 (1.059–1.129)	<0.001	1.072 (1.037–1.109)	<0.001
SOFA score	1.090 (1.046–1.135)	<0.001	0.952 (0.889–1.020)	0.160
Mechanical ventilation				
Patients on mechanical ventilation	2.703 (1.543–4.737)	0.001		
Duration on mechanical ventilation	1.136 (1.071–1.206)	<0.001	1.080 (1.008–1.158)	0.03
Fluid management				
Daily fluid balance (mL/24 h)	1.000 (1.000–1.001)	0.001		
Hemodynamic data				
Duration for MAP < 65 mmHg	1.195 (1.083–1.319)	<0.001	1.149 (1.032–1.279)	0.011
Vasoactive agents				
Vasopressors	1.211 (1.126–1.302)	<0.001	1.082 (0.985–1.188)	0.102
RRT				
Need for RRT *n* (%)	1.825 (1.180–2.822)	0.007		
Time until RRT started (days)	1.261 (1.146–1.388)	<0.001	1.238 (1.115–1.374)	<0.001
Worst KDIGO				
Stage 1 *n* (%)	0.379 (0.216–0.665)	0.001		
Stage 2 *n* (%)	0.625 (0.384–1.019)	0.060		
Stage 3 *n* (%)	2.645 (1.718–4.073)	<0.001	1.466 (0.822–2.613)	0.195
Progress KDIGO class				
Progressive AKI	3.341 (2.039–5.475)	<0.001	3.374 (1.918–5.933)	<0.001

OR: odd ratio; APACHE II score, first 24 h in ICU; SOFA score, first 24 h in ICU.
